# Supplementary material for: Comparison of Methods for Incorporating Related Data When Developing Clinical Prediction Models: A Simulation Study
Source: Biom J. 2026 Aug 27;68(5):e70166. doi: 10.1002/bimj.70166 (PMC13520826; doi:10.1002/bimj.70166)
Supplement: Supplementary file 2 [file BIMJ-68-e70166-s001.zip › third submission code review - R2/CASESTUDY_REALDATA_RESULTS/results/FIGURE7-Performance Metrics of SWEDEHEART Case-Study.pdf]

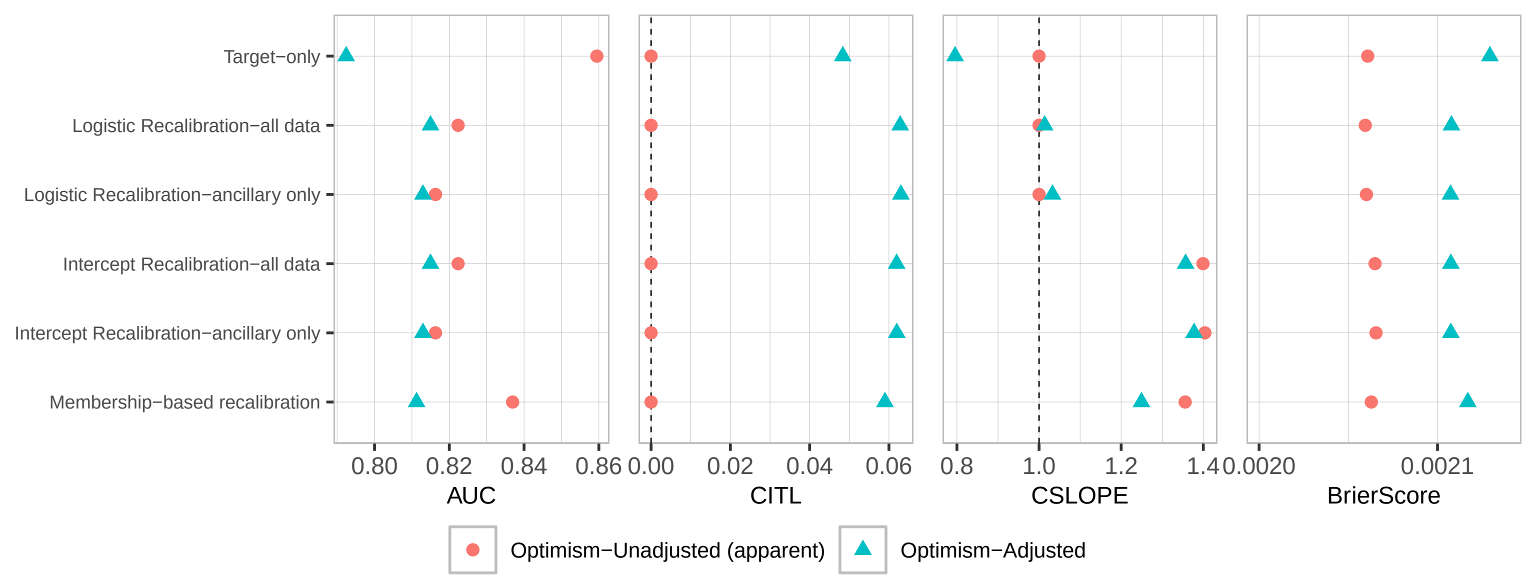

Target−only
Logistic Recalibration−all data
Logistic Recalibration−ancillary only
Intercept Recalibration−all data
Intercept Recalibration−ancillary only
Membership−based recalibration
0.80
0.82
0.84
0.86
AUC
0.00
0.02
0.04
0.06
CITL
0.8
1.0
1.2
1.4
CSLOPE
0.0020
0.0021
BrierScore
Optimism−Unadjusted (apparent)
Optimism−Adjusted
